# Clinical application of single nucleotide polymorphism microarray analysis in pregnancy loss in Northwest China

**DOI:** 10.3389/fgene.2023.1319624

**Published:** 2023-12-14

**Authors:** ShuYuan Xue, LiXia Wang, Jie Wei, YuTong Liu, GuiFeng Ding, PengGao Dai

**Affiliations:** ^1^ The College of Life Sciences, Northwest University, Xi’an, Shanxi, China; ^2^ Prenatal Diagnosis Center, Urumqi Maternal and Child Healthcare Hospital, Urumqi, Xinjiang, China; ^3^ College of Public Health, Xinjiang Medical University, Urumqi, Xinjiang, China; ^4^ Department of Obstetrics, Urumqi Maternal and Child Healthcare Hospital, Urumqi, Xinjiang, China

**Keywords:** single nucleotide polymorphism microarray analysis, chromosome aberrations, copy number variation, pregnancy loss, clinical application

## Abstract

**Background:** Spontaneous abortion is the most common complication of early pregnancy. In this study, we aim to investigate the clinical application value of genetic diagnosis using single nucleotide polymorphism (SNP) microarray analysis on the products of conception and to characterize the types of genetic abnormalities and their prevalence in pregnancy loss in Northwest China.

**Methods:** Over 48 months, we selected 652 products of conception, which included chorionic villi, fetal tissues, germ cell samples, amniotic fluid samples, cord blood samples, and a cardiac blood sample. We analyzed the distribution of chromosomal abnormalities leading to fetal arrest or abortion using SNP array. The patients were then categorized divided into groups based on maternal age, gestational age, number of miscarriages, and maternal ethnic background. The incidences of various chromosomal abnormalities in each group were compared.

**Results:** Of the 652 cases, 314 (48.16%) exhibited chromosomal abnormalities. These included 286 cases with numerical chromosomal abnormalities, 24 cases with copy number variation, and four cases with loss of heterozygosity. Among them, there were 203 trisomy cases, 55 monosomy cases, and 28 polyploidy cases. In the subgroup analysis, significant differences were found in the frequency of numerical chromosomal abnormalities and copy number variation between the advanced and younger maternal age group as well as between the early and late abortion groups. Furthermore, we identified significant differences in the frequency of numerical chromosomal abnormalities between the first spontaneous abortion and recurrent miscarriage groups. However, there were no significant differences in the frequency of numerical chromosomal abnormalities between the Han and Uighur groups.

**Conclusion:** Our research highlights chromosomal abnormalities as the primary cause of spontaneous abortion, with a higher incidence in early pregnancy and among women of advanced age. The use of SNP array analysis emerges as an effective and reliable technique for chromosome analysis in aborted fetuses. This method offers a comprehensive and dependable genetic investigation into the etiology of miscarriage, establishing itself as a valuable routine selection for genetic analysis in cases of natural abortions.

## 1 Introduction

Abortion is the most common adverse pregnancy outcomes; Specifically, spontaneous abortion (SA) occurs in 10%–15% of clinical pregnancies, of which, 1%–5% are recurrent spontaneous abortion events (Rai and Regan, 2006). Pregnant women carrying eggs with genetic abnormalities, and experiencing endocrine dysfunction, prothrombotic predisposition, or advanced age are at a high risk of miscarriage (Campillo et al., 2019). Embryonic chromosomal abnormalities are the most common cause of early SA; 50%–70% of SA events are caused by chromosomal abnormalities (Menasha et al., 2005). Therefore, the detection and analysis of chromosomes in aborted embryonic tissues can help clarify the causes of abortion and provide a basis for the risk assessment of re-pregnancy.

Traditional clinical testing combines chorionic villus cell culture with chromosomal karyotype analysis; however, its cell culture failure rate is high (Shah et al., 2017; Smits et al., 2020). In addition, karyotype analysis can only detect deletions or duplications of more than 5–10 Mb in size, but not chromosomal changes in the submicroscopic structure (Pauta et al., 2018). Although techniques such as fluorescence *in situ* hybridization and real–time quantitative PCR have overcome these shortcomings, their detection range is limited and cannot cover all chromosomes (Shearer et al., 2011). Currently, the detection of products of conception (POCs) has shifted from chromosomal karyotype analysis to genome copy number variation (CNV) analysis (Wang et al., 2020). The commonly used CNV detection platforms in clinical settings include genome CNV analysis based on next-generation sequencing (NGS) and chromosome microarray analysis (CMA) (Clinical Genetics Group Of Medical Genetics Branch Chinese Medical Association, Professional Committee For Prenatal Diagnosis Of Genetic Diseases Medical Genetics Branch Of Chinese Medical Association, Group Of Genetic Disease Prevention, 2019). Copy number variation sequencing (CNV-seq) is an NGS-based method used in most prenatal diagnostic applications. However, CNV-seq fails to detect maternal cell contamination and polyploidy, limiting its application in abortion detection (Clinical Genetics Group Of Medical Genetics Branch Chinese Medical Association, Professional Committee For Prenatal Diagnosis Of Genetic Diseases Medical Genetics Branch Of Chinese Medical Association, Group Of Genetic Disease Prevention, 2019; Wang et al., 2019). CMA includes array-based comparative genomic hybridization (aCGH) and single-nucleotide polymorphism array (SNP array). Both of aCGH and SNP array can detect large-scale and sub-microscopic chromosomal gains and losses. However, aCGH fail to detect polyploidy or maternal cell contamination. The probes of SNP array can determine whether the target sample is contaminated by other samples or parent cells, and can also identify loss of heterozygosity (LOH), single-parent diploids, and triploids (Prenatal Screening And Diagnosis Group Birth Defect Prevention And Control Professional Committee Chinese Preventive Medical Association, 2023; Society for Maternal-Fetal Medicine, 2016).

Although there have been many literature reports on different detection methods to explore the genetic causes of miscarriage tissue (Sahoo et al., 2017; Pauta et al., 2018; Devall and Coomarasamy, 2020; Wang et al., 2020; Chen et al., 2021; Gu et al., 2022), there are no data on genetic testing of pregnancy loss in Northwest China at present. Therefore, it is crucial to explore the genetic etiology of pregnancy loss to provide effective targeted genetic counseling and testing. The purpose of this study was to carry out genetic diagnosis of products of conception in Northwest China and to investigate the types of genetic abnormalities. This effort aims to provide genetic counseling and fertility guidance for couples with adverse pregnancy history, and serve as a reference for prenatal diagnosis and the prevention and control of birth defects. Additionally, we explored the application value of SNP arrays in detecting pregnancy loss in northwest China.

## 2 Materials and methods

### 2.1 Subjects

From January 2019 to December 2022, 660 samples were used for SNP array analysis. Of these, eight cases with poor DNA quality or incomplete clinical information were removed from the primary study, leaving 652 cases available for further investigation. This was a retrospective genetic analysis of a cohort, which included 371 chorionic villi, 266 fetal tissues, nine germ cell samples, three amniotic fluid samples, two cord blood samples, and one cardiac blood samples for SNP array analysis at the Department of Prenatal Diagnosis Center of Urumqi Maternal and Child Health. The gestational age (GA) of the POC samples ranged from 4 to 37 weeks. Maternal age ranged from 21 to 45 years, and the number of miscarriages ranged from one to six, including 180 cases of recurrent miscarriage (RM). RM was defined as two or more miscarriage events. This study was approved by the Ethics Committee of Urumqi Maternal and Child Health Hospital (Approval No. XJFYLL2023037). All POCs were collected under conditions in which couples wanted to unveil the genetic etiology of pregnancy loss. All participants provided written informed consent for genetic investigation involving the detection of fetal chromosomal anomalies using an SNP array.

### 2.2 Sample preparation

POCs were obtained using protocols approved by the institutional review board. Briefly, under aseptic conditions, the sample (either chorionic villi, fetal tissues, or germ cells) was placed in an aseptic vessel and washed with aseptic phosphoate-buffered saline. Genomic DNA was extracted from chorionic villi, fetal tissues, and germs cells using the DNeasy Tissue Kit (Yuanpinghao, Beijing, China), and from amniotic fluid, cord blood, and cardiac blood using the QIAamp DNA Blood Mini Kit (Qiagen, Hilden, Germany), in accordance with the manufacturer’s instructions. A NanoDrop 2000 spectrophotometer (Thermo Fisher Scientific, Madison, GA, United States) was used to determine DNA quantity and quality.

### 2.3 SNP array analysis

SNP array analysis was performed using the Affymetrix CytoScan platform, which included both SNP and copy marker analyses. Genomic DNA underwent a sequential process of digestion, ligation, PCR, PCR product check, purification, quantification, fragmentation, and quality control gel labeling, Finally, the prepared DNA was hybridized to CytoScan 750 K chips, and the resulting arrays were subjected to scanning. Raw data were analyzed using the Affymetrix Chromosome Analysis Suite Software (Affymetrix, Santa Clara, CA, United States), referring to the human assembly GRCh37/hg19. The threshold for the CNV size was set at 500 kb, with at least 50 markers for gains and losses. The chromosomal abnormalities detected by the SNP array were classified into three groups:1) numerical chromosomal abnormalities, including trisomy, monosomy, and polyploidy; 2) large CNVs, i.e., those with gains or losses of chromosome regions >10 Mb in size; and 3) submicroscopic CNVs, i.e., those with gains or losses of chromosome regions <10 Mb in size. Detected CNVs were evaluated based on public databases, including DECIPHER (https://decipher.sanger.ac.uk/search), Database of Genomic Variants (DGV, http://dgv.tcag.ca/dgv/app/home), ClinGen (), UCSC Genome Browser(http://genome.ucsc.edu/cgi-bin/hgGateway), Online Mendelian Inheritance in Man (OMIM, http://www.omim.org/), and PubMed (https://www.ncbi.nlm.nih.gov/pubmed/). CNVs were clinically interpreted according to the American College of Medical Genetics and Genomics guidelines. The chromosomal microdeletions/microduplications were classified as benign CNVs, variants of uncertain significance (VOUS), likely pathogenic CNVs, or pathogenic CNVs (Riggs et al., 2020).

### 2.4 Statistical analyses

Statistical analyses were performed using IBM SPSS software v27.0 (IBM Corp., Armonk, NY, United States). Fisher exact test or Chi-square test was used to analyze differences in CMA yield by different parameters compared to the background factor. Clinical characteristics associated with the occurrence of chromosomal abnormalities using univariate logistic regression analyses, estimating odds ratios and 95% confidence intervals (CIs). Statistical significance was set at *p* < 0.05.

## 3 Results

### 3.1 Overall results

**FIGURE 1:**
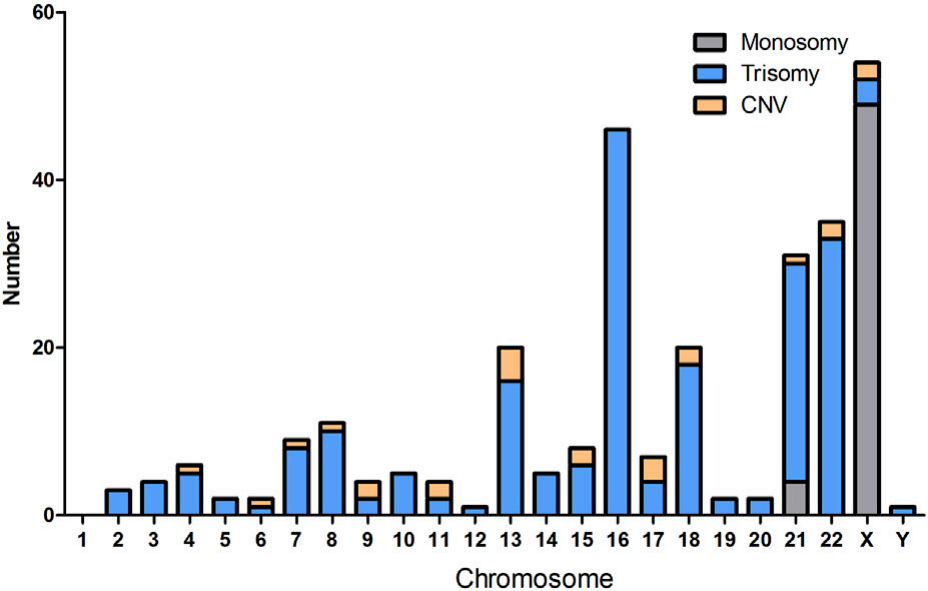
Summary of each chromosome abnormality in fetal specimens of products of conception.

In this study, completely normal results were obtained in 338 cases (338/652, 51.84%). The abnormal results consisted of 286 numerical chromosomal abnormalities (286/652, 43.87%), 24 cases of CNVs (24/652, 3.68%), including 21 pathogenic or likely pathogenic and three VOUS CNVs. In addition, four cases of LOH were detected. Figure 1 shows the details of each chromosomal abnormality observed in this study. The detection rate of numerical chromosomal abnormalities in the advanced maternal age group (≥35 years old) was 64.81% (70/108), higher than that of the younger maternal age group (<35 years old), which had a rate of 39.71% (216/544). The numerical chromosomal abnormality rate in the early abortion group (GA <12 weeks) was 56.38% (212/376), higher than that of the late abortion group (GA ≥12 weeks), which had a rate of 26.81% (74/276). The numerical chromosomal abnormality rate in the RM group was 51.11% (92/180), higher than that of the first spontaneous abortion (FSA) group, which had a rate of 41.10% (194/472). The rate of numerical chromosomal abnormalities in the Han population group was 44.19% (255/577), similar to that of the Uyghur group, which had a rate of 41.33% (31/75). Significant differences were found between the numerical chromosomal abnormalities and the normal chromosome groups in terms of maternal age (*p* < 0.001), GA (*p* < 0.001), and number of miscarriages (*p* = 0.014). Significant differences were found between the CNVs and normal chromosome groups in terms of maternal age (*p* = 0.04) and GA (*p* = 0.04). However, no significant differences were found between the numerical chromosomal abnormalities/CNVs and the normal chromosome groups with respect to maternal ethnic background. The detailed results are presented in Table 1.

**TABLE 1:** Single nucleotide polymorphism microarray analysis results of products of conception in 652 cases [n(%)].

Detection result	No. (case)	Maternal age (years)		χ^2^	*p*	GAs (weeks)		χ^2^	*p*	Number of miscarriage		x2	*p*	Maternal ethnic background		χ^2^	*p*
		<35	≥35			<12	≥12			FSA	RM			Han	Uighur		
Numerical chromosomal abnormalities	286	216 (39.71%)	70 (64.81%)	24.43	<**0.001**	212 (56.38%)	74 (26.81%)	60.44	<**0.001**	194 (41.10%)	92 (51.11%)	6.01	**0.014**	255 (44.19%)	31 (41.33%)	0	1
CNV	24	18 (3.31%)	6 (5.56%)	4.22	**0.04**	16 (4.26%)	8 (2.90%)	4.21	**0.04**	15 (3.18%)	9 (5%)	1.82	0.177	18 (3.12%)	6 (8%)	3.21	0.073
LOH	4	4 (0.74%)	0	0	1	3 (0.80%)	1 (0.36%)	0.61	0.44	3 (0.64%)	1 (0.56%)	0	1	2 (0.35%)	2 (2.67%)	2.85	0.091
Normal	338	306 (56.25%)	32 (29.63%)	-	-	145 (38.56%)	193 (69.93%)	-	-	260 (55.08%)	78 (43.33%)	-	-	302 (52.34%)	36 (48%)	-	-
Total (case)	652	544	108	-	-	376	276	-	-	472	180	-	-	577	75	-	-

Data are presented as number and percentage for every group. Fisher’s exact analyses, *p* < 0.05 vs. Normal; Significant *p*-values (<0.05) are highlighted in bold. Abbreviation: No., numbers; GAs, gestational ages; FSA, first spontaneous abortion; RM, recurrent miscarriage; CNV, copy number variation; LOH, loss of heterozygosity.

### 3.2 Spectrum of chromosomal number abnormalities

Chromosomal number abnormalities were the most frequently-detected chromosomal aberrations. These aberrations were identified in approximately 91.08% of cases (286/314), including 203 trisomy, 55 monosomy, and 28 polyploidy cases. All polyploidy tests were performed in the triploid form. Trisomy 16 was the most frequent trisomy (46/203, 22.66%), followed by trisomy 22 (33/203, 16.26%). The incidence of trisomy was higher in the advanced maternal age group (60/108, 55.56%) than in the younger maternal age group (143/544, 26.27%), and the difference was statistically significant (*p* < 0.001). The incidences of trisomy, monosomy, and polyploidy were higher in the early abortion group than in the late abortion group (*p* < 0.001, *p* = 0.007, and *p* < 0.001, respectively). The incidence of trisomy and polyploidy was higher in the RM group than in the FSA group (*p* = 0.029 and *p* = 0.035, respectively). The detailed results are presented in Table 2.

**TABLE 2:** Distribution of numerical chromosomal abnormalities in 286 products of conception detected with single nucleotide polymorphism microarray analysis [n(%)].

Grouping of numerical chromosomal abnormalities	No. (case)	Maternal age (years)		χ^2^	*p*	GAs (weeks)		χ^2^	*p*	Number of miscarriage		χ^2^	*p*	Maternal ethnic background		χ^2^	*p*
		<35	≥35			<12	≥12			FSA	RM			Han	Uighur		
Trisomy	203	143 (66.20%)	60 (85.71%)	34.86	<0.001	155 (73.11%)	48 (64.86%)	56.12	<0.001	138 (71.13%)	65 (70.65%)	4.77	**0.029**	178 (69.80%)	25 (80.65%)	0.21	0.651
Monosomy	55	48 (22.22%)	7 (10%)	0.26	0.612	35 (16.51%)	20 (27.03%)	7.38	**0.007**	40 (20.62%)	15 (16.30%)	0.26	0.612	50 (19.61%)	5 (16.13%)	0.01	0.91
Polyploidy	28	25 (11.57%)	3 (4.29%)	0	1	22 (10.38%)	6 (8.11%)	11.86	<0.001	16 (8.25%)	12 (13.04%)	4.44	**0.035**	27 (10.59%)	1 (3.23%)	0.75	0.385
Total (case)	286	216	70	-	-	212	74	-	-	194	92	-	-	255	31	-	-
Normal	338	306	32	-	-	145	193	-	-	260	78	-	-	302	36	-	-

Data are presented as number and percentage for every group. Fisher’s exact analyses, *p* < 0.05 vs. Normal; Significant *p*-values (<0.05) are highlighted in bold. Abbreviation: No., numbers; GAs, gestational ages; FSA, first spontaneous abortion; RM, recurrent miscarriage.

### 3.3 Chromosomal microdeletions and microduplications

Chromosomal microdeletions and microduplications were detected in 24 cases, with the largest fragment being 116.11 Mb in size and the smallest fragment being 1.32 Mb in size. In total, 26 pathogenic CNVs and three likely pathogenic CNVs were detected in 21 cases. Four VOUS CNVs were identified in three cases. There were 15 pathogenic or likely pathogenic CNVs microdeletions, and 14 pathogenic or likely pathogenic microduplications (Table 3).

**TABLE 3:** Copy number variants found by single nucleotide polymorphism microarray analysis in products of conception.

ID	MA (years)	GAs (weeks)	MEB	NM	Fetal ultrasound	SNP array result	Size(Mb)	Interpretation	Related syndrome
2	35	23	U	1	Polyhydramnios	18p11.32(558353-2262415)x3	1.70	VUS	-
20	28	10	H	1	N	6q27(166973398-170914297)x1	3.94	P	-
40	39	9	H	2	N	13q31.1q34(84755591-115107733)x3	30.35	P	-
50	29	11	H	2	N	17p13.3p13.1(525-7575114)x3	7.57	P	Split-hand/foot malformation with long bone deficiency 3 syndrome
						13q14.3q34(51399757-115107733)x1	63.71	P	Microcoria, congenital (chromosome 13q32 deletion) syndrome
121	42	9	H	1	N	8p23.1p22(12532774-16761578)x3	4.23	VUS	-
						8p23.3p23.1(158049-6999220)x1	6.84	VUS	-
211	36	6	U	2	N	11p15.5q11(564114-116677848)x3	116.11	P	Spinocerebellar ataxia 20 (chromosome 11q12 duplication syndrome, 260 kb) syndrome
						22q11.1q11.21(16888900-20312661)x1	3.42	P	22q11 deletion syndrome (Velocardiofacial/DiGeorge syndrome)
233	31	11	H	2	N	9p24.3q21.12(208455-73187232)x3	72.98	P	-
						15q11.2q14(22770422-34944224)x1	12.17	P	Prader-Willi syndrome (Type 1)/Angelman syndrome (Type 1)
261	31	9	H	2	N	4q33q35.2(171499063-190957460)x1	19.46	LP	-
						6p25.3p22.1(156975-28542845)x3	28.39	LP	-
270	31	12	H	1	N	18p11.32p11.21(136228-15170636)x1	15.03	P	-
296	28	9	H	1	N	15q26.1q26.3(90268768_102429040)x1	12.16	P	Chromosome 15q26-qter deletion syndrome
						21q22.2q22.3(41242192_48093361)x3	6.85	P	Down syndrome chromosome region
312	29	24	H	1	Digestive tract malformation	15q11.2q13.1(22770422_28522492)x3	5.75	P	15q11.2q13 recurrent (PWS/AS) region (Class 1, BP1-BP3) duplication syndrome
316	30	16	H	1	Spina bifida	22q11.21(18648856_21800471)x1	3.15	P	22q11 deletion syndrome (Velocardiofacial/DiGeorge syndrome)
355	28	8	H	1	N	Xp22.33p22.32(168552_4531210)x1	4.36	P	-
						11q24.1q25(122813871_134937416)x3	12.12	LP	-
403	23	20	U	1	Ventricular enlargement	13q31.1q34(84031141_115107733)x1	31.08	P	Microcoria, congenital (chromosome 13q32 deletion) syndrome
427	32	11	H	2	N	Xq21.1q28(83251922_155233098)x1	71.98	P	-
458	26	8	H	1	N	17q12(34449166_36243365)x3	1.79	P	17q12 recurrent (RCAD syndrome) region (includes HNF1B) duplication
506	28	8	H	1	N	17q23.2q25.3(60404980_81041823)x3	20.64	P	46XX sex reversal 2
						9q34.3(139694476_141018648)x1	1.32	P	9q subtelomeric deletion syndrome
557	24	8	U	2	N	7q34q36.3(138687691_159119707)x1	20.43	P	-
						11p15.5p15.1(230681_17221755)x3	16.99	P	-
559	29	7	U	4	N	9p24.3p13.1(208455_38772005)x4	38.56	P	-
571	24	24	H	1	Lower extremity deformities, kidney size inconsistencies	11q22.1q25(99610284_134937416)x3	35.33	P	-
598	35	10	H	1	N	13q31.1q31.2(84639529_87806179)x1	3.17	VUS	-
601	36	9	H	2	N	21q21.3q22.3(30354632_48093361)x3	17.74	P	Down syndrome chromosome region
608	28	33	U	1	N	22q11.21q11.23(21804597_24659578)x1	2.86	P	22q11.2 distal deletion syndrome
641	29	22	H	1	Neural tube defects, whole forebrain	13q31.2q34(88053157_115107733)x1	27.06	P	Microcoria, congenital (chromosome 13q32 deletion) syndrome

Abbreviation: MA, maternal age; GAs, gestation ages; MEB, maternal ethnic background; H, Han; U, Uighur; N, normal; NM, Number of miscarriage; P, pathogenic; LP, likely pathogenic; VUS, variants of unclear clinical significance.

### 3.4 Risk factors associated with miscarriage

Logistic regression models were used for calculating the odds ratios (95% confidence intervals) and corresponding *p*-values for the association of clinical characteristics with the occurrence of chromosomal abnormalities in spontaneous abortion specimens. Upon carrying out single factor regression, the *p*-values for the continuous variables–maternal age, and gestational age were less than 0.05 (Table 4).

**TABLE 4:** Logistic regression of clinical characteristics with chromosomal abnormalities occurrence.

Characteristics	*p*-value	OR	95% CI
Maternal age	0.001	1.071	1.027-1.117
Gestational age	<0.001	0.879	0.850-0.910
Number of miscarriages	0.664	1.06	0.816-1.376
Maternal ethnic background	0.148	0.674	0.395-1.150

Abbreviation: OR, odds ratios; 95% CI, 95% confidence interval.

## 4 Discussion

Pregnancy loss is a multifactorial disorder. Currently, the causes and mechanisms of SA are mainly genetic, immune, thrombolysis-induced, anatomical, and endocrine factors. Chromosomal abnormalities in embryos are a common cause of SA, with the incidence of chromosomal abnormalities in embryos that have stopped developing exceeding 50%, in embryos that have ceased development. These abnormalities include variations in chromosome numbers and fragment duplications/deletions (Rai and Regan, 2006; Nikitina et al., 2020). Recent studies have shown that multiple chromosome microduplications and microdeletions may contribute to SA by affecting pregnancy-related genes or pathways (Bagheri et al., 2015; Pauta et al., 2018; Wang et al., 2020; Gu et al., 2022).

Currently, aCGH, SNP array, or CNV-seq technology platforms can be used to detect genomic CNVs in POCs. However, both aCGH and CNV-seq fail to detect polyploidy or maternal cell contamination (Prenatal Screening And Diagnosis Group Birth Defect Prevention And Control Professional Committee Chinese Preventive Medical Association, 2023; Chen et al., 2021; Genetic Disease Prevention And Control Group Of Professional Committee For Birth Defect Prevention And Control Of Chinese Preventive Medicine Association, Clinical Genetics Group Medical Genetics Branch Of Chinese Medical Association, 2023). SNP array technology can not only detects numerical chromosomal abnormalities and chromosome microduplication/microdeletion but can also detects LOH and uniparental diploidy (Zhang et al., 2018; Qu et al., 2019). The SNP array incorporates genome-wide SNP typing probes unlike conventional platforms. SNP array can be used to differentiate between alleles or haplotypes, to detect ploidy changes in chromosomes, and display distinct signal line profiles based on the principle that haplotypes of the fetus differ from those of the mother in samples with maternal contamination. The SNP probes are designed to help detect contamination of maternal cells in POCs, eliminating the need for the short tandem repeat test for samples in POC samples. In addition, for CNV analysis, the double validation of both the CNV probe and the SNP probe in the SNP array ensures the accuracy of copy number variation detection, which has been confirmed in several studies (Shah et al., 2017; Babu et al., 2018; Smits et al., 2020). While the SNP array offers numerous advantages, it shares some limitations with aCGH and CNV-seq. These limitations include the inability to detect chromosome balance translocations, inversions, complex rearrangements, single nucleotide variations, and low-proportion chimerism. Nonetheless, SNP arrays still have the highest detection rate of abnormal chromosomes in SA tissue (Smits et al., 2020). The application of SNP arrays has enhanced the diagnostic precision of SA, providing valuable insights for assessing the risk of miscarrying in pregnant women. In total, 652 specimens were examined in this study, revealing an overall detection rate of chromosomal abnormality detection rate of 48.16% (314/652). Numerical chromosomal abnormalities constituted the majority, accounting for approximately 91.08% of cases (286/314).

Among of the numerical chromosomal abnormalities, trisomy was the most common abnormality (203/314, 64.65%), followed by monosomy (55/314, 17.52%), and polyploidy (28/314, 8.92%). Trisomy and monosomy are usually caused by the non-separation of a certain chromosome during gamete formation in one of the parents or during zygote cleavage in early pregnancy, leading to an increase or decrease in one chromosome. In this study, the proportion of trisomy 16 was the highest (46/204, 22.55%), and trisomies 8, 13, 18, 21, and 22 also accounted for a large proportion. We detected all autosomal trisomies except for in chromosome 1 in the POCs, consistent with previous reports (Tekcan et al., 2015; Shen et al., 2016; Sahoo et al., 2017; Wang et al., 2017; Pauta et al., 2018). The proportion of X monomer was the highest among all monomers (49/55, 89.09%). Studies have shown that all trisomy 16 abortions occur in early pregnancy, and a small number of trisomy 15,18,21 and X monomer embryo abortions can occur in the second trimester or late pregnancy (Warren and Silver, 2008). In our study, the GAs of trisomy 16 were all within 14 weeks, the GAs of monosomy X were all within 17 weeks, and the GAs of trisomies 15, 18, and 21 ranged from 7 to 26 weeks. Autosomal trisomy results from chromosome nondissociation in the late stages of meiosis and cell division. Theoretically, the probability of nondissociation of all chromosomes is equal. However, Hassold’s studies showed that the nondissociation rate differed among chromosomes, and chromosome 16 had the highest nondissociation rate (Hassold and Jacobs, 1984). Chromosome 16 is one of the most abundant segmental duplications sequences (SD) in the genome. The SD sequences were mainly concentrated in 16p, with the largest region located in the 16p11 region, and this structural specificity resulted in a high incidence of genome copy number variation in chromosome 16 (Martin et al., 2004). This may be the reason why chromosome 16 has a relatively high probability of chromosome non-separation during cell division. Trisomy is sporadic and occurs occasionally during germ cell formation. If one or both spouses are of advanced age, or have been exposed to teratogenic drugs, radiation, adverse environmental conditions, etc., there may be an increased the risk of pregnancy with a trisomic fetus. Therefore, our study demonstrates the importance of chromosomal examination of aborted embryos in identifying genetic causes. Couples who have experienced the birth of trisomic fetuses should pay attention to avoid exposure to toxic and harmful substances in the environment before or during the next pregnancy, and prenatal diagnostic testing should be performed during the subsequent pregnancy.

CNVs play an important role in prenatal ultrasound abnormalities and neurodevelopmental disorders (Deshpande and Weiss, 2018; Levy and Wapner, 2018). In our study, we detected CNVs in 24 patients (24/652,3.68%). This detection rate for submicroscopic CNVs is consistent with previous report (Zhu et al., 2018). We found 26 pathogenic CNVs, three likely pathogenic CNVs, and four various CNVs. Additionally, we identified 10 cases with pCNVs less than 10 Mb, which would not have been detectable with G-banding karyotyping. Pathogenic CNVs identified in our study encompassed various syndromes, including 22q11.2 microdeletion, chromosome 13q32 deletion syndrome, 9q subtelomeric deletion syndrome, chromosome 15q26-qter deletion syndrome, 17q12 recurrent (RCAD syndrome) region duplication, 46XX sex reversal 2 syndrome, chromosome 11q12 duplication syndrome, and 15q11.2q13 recurrent (Prader-Will syndrome/Angelman syndrome) region (Class 1, breakpoints (BP): BP1–BP3) duplication. Some of these cases have also been reported in other abortion reports (Liu et al., 2015; Wang et al., 2017; Pauta et al., 2018). However, it remains unclear whether these deletions and duplications lead to miscarriages. Although some studies have compared the prevalence of CNVs in POCs within the general population and suggested an association with pregnancy loss, no definitive conclusions have been made due to a lack of evidence. In our cohort, the results indicated that three samples carried four VOUS CNVs that did not involve geneswith critical roles in embryonic development. However, the parents refused SNP array testing to clarify the source of these CNVs.

SNP arrays allow the identification of polyploidy and LOH (Singh et al., 2013). Polyploidy was reported in 4.44%–8.8% of miscarriage cases carried polyploidy in the published literature (Singh et al., 2013; Sahoo et al., 2017). In this study, the rate of pregnancy loss cases with polyploidy detected by SNP array was 4.29% (28/652), consistent with previous reports. As previously reported, the incidence of uniparental disomy was 0.24%–1.9% in products of miscarriage (Singh et al., 2013; Liu et al., 2015; Sahoo et al., 2017). Notably, the frequency of uniparental disomy is likely to be higher because CNV-seq and traditional cytogenetic technologies cannot detect LOH. In the present cohort, LOH was identified in four samples (4/652, 0.61%), affecting chromosomes 1, 5, 20, and X, none of which were associated with genetic imprinting. The presence of LOH due to uniparental disomy or parental consanguinity was not determined, as it was at the patient’s discretion.

Age is a high risk factor for chromosomal abnormalities. The quality of female oocytes decreases with age, leading to a notable increase in the incidence of aneuploidy (Qu et al., 2019; Huang et al., 2022). Fetal chromosomal abnormalities are the leading causes of miscarriages, particularly during the early stages of pregnancy. Therefore, the numbers of miscarriage and GA are also considered interrelated factors (Rai and Regan, 2006; Ticconi et al., 2016; Devall and Coomarasamy, 2020). In the subgroup analysis, the results were classified based on maternal age, gestational age, number of miscarriages, and maternal ethnic background. In this study, the detection rate of abnormal chromosome number and structure in the ≥35 years group was higher than that in the <35 years group (*p* < 0.001 and *p* = 0.04, respectively). Meanwhile, the <35 years group in this study also had a high detection rate of abnormal staining numbers and structural abnormalities. Therefore, screening for chromosomal number and structural abnormalities should include pregnant women in all age groups. Further analysis suggests that the detection rate of chromosomal number and structural abnormalities in the <12 weeks pregnant group is higher than that in the ≥12 weeks group (*p* < 0.001 and *p* = 0.04, respectively). The above results are similar to those in previous reports (Liu et al., 2015; Wang et al., 2017; Pauta et al., 2018). The detection rate of chromosomal abnormalities was higher in the RM group than in the FSA group (*p* = 0.0014); however, there was no significant difference in chromosomal structural abnormalities. Based on regional data, we found no significant differences in chromosome number or structural abnormalities between the Han and Uyghur populations. In addition, our results showed that the incidence of trisomy in pregnancy loss was associated with maternal age, GA, and the number of miscarriages (*p* < 0.001, *p* < 0.001, and *p* = 0.029, respectively). However, the analysis did not find a significant correlation with maternal age. Meanwhile, the incidence of monosomy in pregnancy loss was only associated with GA (*p* = 0.007) but not with maternal age, frequency of miscarriage, or maternal ethnic background. The incidence of polyploidy in pregnancy loss was associated with GA and frequency of miscarriage (*p* < 0.001 and *p* = 0.035, respectively), but was not associated with maternal age or ethnic background.

## 5 Conclusion

In conclusion, we evaluated the feasibility of SNP analysis in clinical practice for analyzing chromosomal abnormalities in natural abortion specimens. Our results confirm that chromosomal abnormalities are the most common cause of pregnancy loss, and that maternal age, GA, and number of miscarriages are related to fetal chromosomal abnormalities. The SNP array proves to be a reliable method for evaluating the genetic etiology of pregnancy loss, enabling a relatively comprehensive genetic analysis of miscarriage villous tissue and identification of various genetic factors that cause miscarriage. Couples experiencing pregnancy loss might be advised to undergo genetic analysis using SNP arrays, guiding the selection of reproduction methods and prenatal diagnosis to prevent the recurrence of abortion and the birth of children with chromosomal diseases.

## Data Availability

The datasets presented in this article are not readily available due to privacy concerns. Requests to access the datasets should be directed to the corresponding author.

## Abbreviations

Single nucleotide polymorphism, (SNP); spontaneous abortion, (SA); products of conception, (POCs); copy number variation, (CNV); next-generation sequencing, (NGS); chromosome microarray analysis, (CMA); comparative genomic hybridization, (aCGH); Copy number variation sequencing, (CNV-seq); loss of heterozygosity, (LOH); gestational age, (GA); recurrent miscarriage, (RM); variants of uncertain significance, (VOUS); first spontaneous abortion, (FSA); segmental duplications sequences, (SD).
